# LMA Gastro™ airway is feasible during upper gastrointestinal interventional endoscopic procedures in high risk patients: a single-center observational study

**DOI:** 10.1186/s12871-020-0938-9

**Published:** 2020-02-08

**Authors:** Axel Schmutz, Thomas Loeffler, Arthur Schmidt, Ulrich Goebel

**Affiliations:** 1 Department of Anesthesiology and Critical Care, Faculty of Medicine, Medical Center - University of Freiburg, University of Freiburg, Hugstetter Strasse 55, 79106 Freiburg im Breisgau, Germany; 2 Department of Medicine II, Faculty of Medicine, Medical Center - University of Freiburg University of Freiburg, Hugstetter Strasse 55, Freiburg im Breisgau, 79106 Germany

**Keywords:** Achalasia, Endoscopic procedures, Endoscopic submucosal dissection, High-risk patients, Supraglottic airway device

## Abstract

**Background:**

Nonoperating room anesthesia during gastroenterological procedures is a growing field in anesthetic practice. While the numbers of patients with severe comorbidities are rising constantly, gastrointestinal endoscopic interventions are moving closer to minimally invasive endoscopic surgery. The LMA Gastro™ is a new supraglottic airway device, developed specifically for upper gastrointestinal endoscopy and interventions. The aim of this study was to evaluate the feasibility of LMA Gastro™ in patients with ASA physical status ≥3 undergoing advanced endoscopic procedures.

**Methods:**

We analyzed data from 214 patients retrospectively who received anesthesia for gastroenterological interventions. Inclusion criteria were upper gastrointestinal endoscopic interventions, airway management with LMA Gastro™ and ASA status ≥3. The primary outcome measure was successful use of LMA Gastro™ for airway management and endoscopic intervention.

**Results:**

Thirtyone patients with ASA physical status ≥3, undergoing complex and prolonged upper gastrointestinal endoscopic procedures were included. There were 7 endoscopic retrograde cholangiopancreatographies, 7 peroral endoscopic myotomies, 5 percutaneous endoscopic gastrostomies and 12 other complex procedures (e.g. endoscopic submucosal dissection, esophageal stent placement etc.). Of these, 27 patients were managed successfully using the LMA Gastro™. Placement of the LMA Gastro™ was reported as easy. Positive pressure ventilation was performed without difficulty. The feasibility of the LMA Gastro™ for endoscopic intervention was rated excellent by the endoscopists. In four patients, placement or ventilation with LMA Gastro™ was not possible.

**Conclusions:**

We demonstrated the feasibility of the LMA Gastro™ during general anesthesia for advanced endoscopic procedures in high-risk patients.

**Trial registration:**

German Clinical Trials Register (DRKS00017396) Date of registration: 23rd May 2019, retrospectively registered.

## Background

Nonoperating room anesthesia in the gastroenterology suite is a growing field in anesthesiology practice [1, 2]. While the majority of gastrointestinal endoscopies are performed under conscious sedation by non-anesthesia personnel, there is a shift towards deep sedation or general anesthesia for advanced procedures and interventions [3]. Especially for patients with ASA physical status ≥3, high BMI, obstructive sleep apnea and severe comorbidities, the presence of an anesthesiologist is recommended [3–5]. General anesthesia is associated with shorter procedure times, higher complete resection rates, a decreased incidence of coughing and lower perforation rates [6–8]. From a practical point of view, securing an airway for complex procedures with a gastroscope in situ usually requires tracheal intubation. The numbers of patients with severe comorbidities presenting for upper gastrointestinal endoscopic interventions are rising, so the need for a fast and yet gentle and safe airway device is relevant.

Dual channel laryngeal masks, also referred to as “second-generation” supraglottic airway devices (2nd SAD), are defined by the presence of an accessory tube for gastric drainage. Shortly after the first commercially available 2nd SAD [9], there was a report of a modified, self-made laryngeal mask, which allowed passing instruments like a gastroscope through the accessory tube into the esophageus [10]. A modified laryngeal tube, the gastro-laryngeal tube (VBM Medizintechnik GmbH, Sulz am Neckar, Germany), has been described as an alternative airway device with a dedicated channel for an endoscope [11]. This approach was not developed further until 2017, when M. Skinner introduced a refined tool in advanced airway management for upper gastrointestinal endoscopy (i.e. the LMA® Gastro™ Airway^1^) [12, 13]. This silicone-based, cuffed LMA offers an additional and separate channel for the passage of instruments (such as an endoscope) of up to a width of 16 mm in diameter [14]. If the mask is placed correctly the endoscope is guided directly towards the upper oesophageal entrance and the airway is left unobstructed. Ventilatory parameters, especially peak airway pressure, are not altered due to insertion of the endoscope through the separate channel. The endoscope may glide easily through the channel, providing excellent conditions for the endoscopist in terms of endoscope movement, including rotation and interventions. To date, the new LMA Gastro™ Airway has only been evaluated in healthy patients for diagnostic upper gastrointestinal endoscopy, demonstrating good efficacy without any detrimental or harmful side effects [12].

It was the aim of this study to evaluate the feasibility of this tool not only in ASA I and II patients for diagnostic upper gastrointestinal endoscopy, who may not even require general anesthesia but in older and high risk patients, undergoing more complex and lengthy endoscopy and subsequently more challenging interventions under general anesthesia.

## Methods

This retrospective cohort analysis was approved by the local Ethics Committee, University of Freiburg, Germany (approval number EK 37/19, date: March 19th 2019, PI: Axel Schmutz, MD). The study was conducted at the Department of Anesthesiology and Critical Care and the Department of Medicine II, Medical Centre – University of Freiburg, Faculty of Medicine, Freiburg, Germany. The study was registered in the German Clinical Trials Register (DRKS00017396), PI Axel Schmutz, MD, on May 23rd 2019. The study was planned and designed in accordance with the initiative for Strengthening the Reporting of Observational Studies in Epidemiology (STROBE), using the suggested checklist for epidemiological cohort studies [15]. The data of closed files between October 2018 and March 2019 was collected by chart review and entered into a database. 214 patients who underwent upper gastrointestinal endoscopic procedures and interventions were included; 31 patients were finally analyzed. Written informed consent was obtained from all subjects, a legal surrogate, or the parents or legal guardians. A priori sample size calculation was not applicable due to the retrospective study design.

High-risk patients undergoing elective upper gastrointestinal endoscopic procedures and interventions with the need for general anesthesia were analyzed. Inclusion criteria were airway management with LMA Gastro™, ASA status 3 or above, age 10 years and older, body weight above 30 kg. Patients fasted for at least 6 h for solids and at least two hours for clear fluids. It was our intention not to exclude any patients with esophageal reflux, esophageal strictures, cancer of the upper gastrointestinal tract or the stomach, achalasia, pancreatitis, upper GI bleeding, colitis, pyloric stenosis, morbid obesity etc.

### Anesthetic management

After implementation of routine monitoring (ECG, non-invasive blood pressure, peripheral oxygen saturation) general anesthesia was induced using i.v. sufentanil (0.2–0.3 μg·kg^− 1^ body weight (BW)) followed by remifentanil (0.1–0.5 μg ·kg^− 1^·min^− 1^) and propofol 3.0–5.0 μg·ml^− 1^ effect site concentration target-controlled infusion (Agilia™, Schnider model). Paralytic medication was not administered. Normocapnia and normoxia were achieved by positive pressure ventilation (pressure controlled ventilation, positive endexspiratory pressure: of 3–5 mbar, respiratory rate: 10–18/min) with a F_I_O_2_ of 0.5 after insertion of the lubricated LMA Gastro™ Airway (Size 3, 4 or 5). The LMA cuff was filled with air until the indicator of the cuff pressure valve was in the “green zone” of the cuff pressure valve. This corresponds to a cuff pressure of 40 cm H_2_O. (Cuff Pilot, embedded cuff monitoring system). According to our departmental standards a successful insertion was assumed by a square wave pattern capnography, symmetrical chest wall expansion and absence of an audible oral air leak with a cuff pressure between 20 and 40 cmH_2_O. Haemodynamic shifts (mean arterial blood pressure ± 20% regarding baseline values) were treated with i.v. ephedrine/norepinephrine or urapidil respectively. After fixation of the LMA Gastro™ Airway, a well lubricated flexible endoscope was inserted into the gastric channel and advanced into the oesophagus. Anesthetic and endoscopic records and ward charts of patients were analyzed for periinterventional data regarding airway management, pharyngeal bleeding, sore throat, hoarseness and any other serious adverse events during or within 24 h after the endoscopic intervention.

If tracheal intubation was necessary, neuromuscular block was induced using an i.v. bolus of rocuronium bromide (0.6–1.0 mg·kg^− 1^ body weight).

The combined outcome was defined as the feasibility to use the LMA Gastro™ successfully for both, the endoscopic intervention and a sufficient airway management in the defined high-risk patients. Secondary outcome parameters comprised (a) the ease of placement of the LMA Gastro™ or any additional attempts required to correctly place the device, (b) the need for any alternative airway devices (i.e. tracheal tube) in case of uncontrollable permanent airway leakage, (c) any form of dislocation associated with the endoscopic procedure, thus necessitating an alternative airway tool, other than the LMA Gastro™, (d) the incidence of pharyngeal bleeding during placement or after the removal of the LMA Gastro™, (e) any unwanted events during the endoscopic procedure with the main focus on regurgitation, aspiration or hypoxia, (f) duration of the endoscopic procedure, (g) sore throat and/or hoarseness and (h) the comfort of advancing and operating the endoscope through the gastric channel rated by the attending endoscopist after the procedure via a 5 point Likert-type scale (0 = not at all satisfied, 1 = slightly satisfied, 2 = moderately satisfied, 3 = very satisfied, 4 = completely satisfied).

The data was collected in a MS Excel™ (Microsoft, Redmond, USA) datasheet. Further statistical processing was performed using SPSS™ (IBM, Armonk, USA).

## Results

**Fig. 1:**
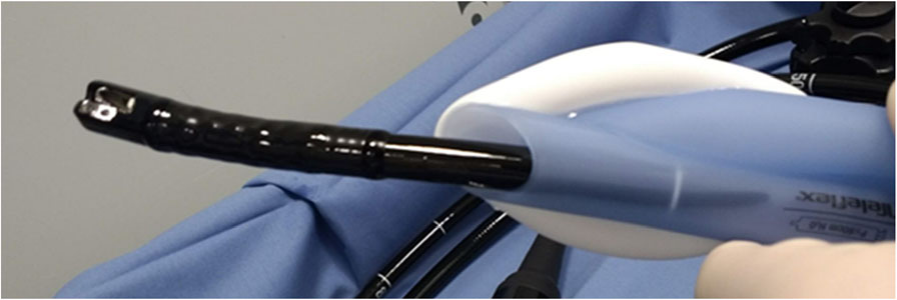
Gastroduodenoscope passing through the gastric channel of a LMA Gastro™

Of 214 patients receiving anesthetic care in the gastroenterology suite, 75 patients were ventilated with the LMA Gastro™. Thirtyone cases were advanced procedures in high-risk patients lasting for a median of 60 min (Additional file 1: Figure S1). Patient characteristics, endoscopic interventions, duration of the procedures and the endoscopists’ ratings are summarized in Table 1. These 31 endoscopic interventions were performed under general anesthesia, of which 27 finally were performed successfully with LMA Gastro™ (Fig. 1).

**Table 1:** Patient characteristics, endoscopic procedures (*n* = 31). Values are number (proportion) or median (IQR [range])

Age (yrs) median [IQR]	65 [31–72]
Sex	
male	23
female	8
ASA physical status^*a*^	
3	22 [71%]
4	9 [29%]
Body Mass Index median [IQR]	24 [20–25]
Reflux	9 [32%]
Results	
Endoscopic procedure	
PEG^*b*^	5 [16%]
ERCP^*c*^	7 [23%]
POEM^*d*^	7 [23%]
ESD^*e*^, STER^*f*^	6 [19%]
other	6 [19%]
Ventilation and endoscopic procedure through LMA Gastro™ successful	27 [87%]
Duration of endoscopic procedure (min) median, [IQR]	60 [25–75]
Sore throat and/or hoarseness (in PACU)	4 [13%]
Rating of endoscopist^*g*^, mean [range]	3.7 [3–4]

Abbreviations: ^*a*^ASA physical status: ASA Physical Status Classification System [16]; ^*b*^PEG, percutaneous endoscopic or fluoroscopic gastrostomy; ^*c*^ERCP, endoscopic retrograde cholangiopancreatography; ^*d*^POEM, peroral endoscopic myotomy; ^*e*^ESD, Endoscopic submucosal dissection; ^*f*^STER, submucosal tunneling endoscopic resection. ^*g*^Rating of endoscopist Likert-type scale (0 = not at all satisfied, 1 = slightly satisfied, 2 = moderately satisfied, 3 = very satisfied, 4 = completely satisfied)

**Fig. 2:**
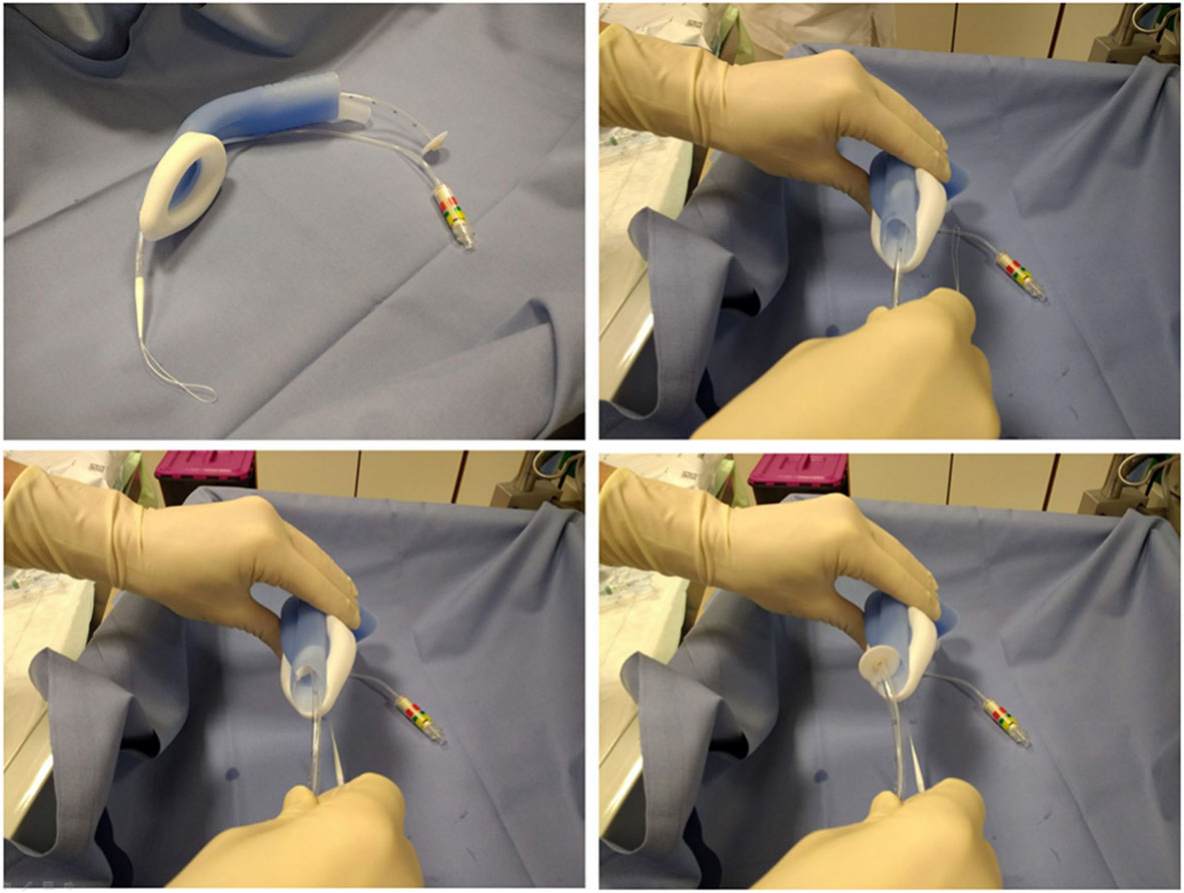
Percutaneous endoscopic gastrostomy, “pull” technique

**Fig. 3:**
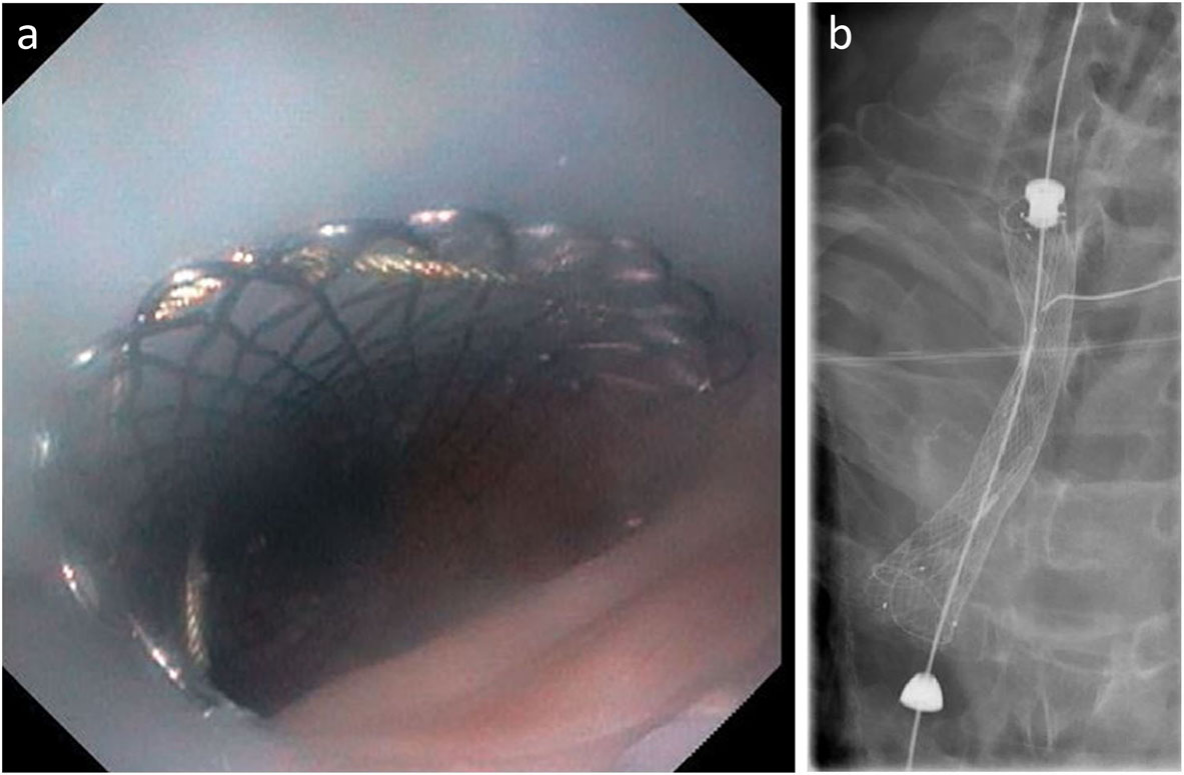
Esophageal stent placement. **a** Endoscopic view through the gastric channel of a LMA Gastro™. **b** Chest X-ray: Esophageal stent placement, LMA Gastro™ in situ

**Fig. 4:**
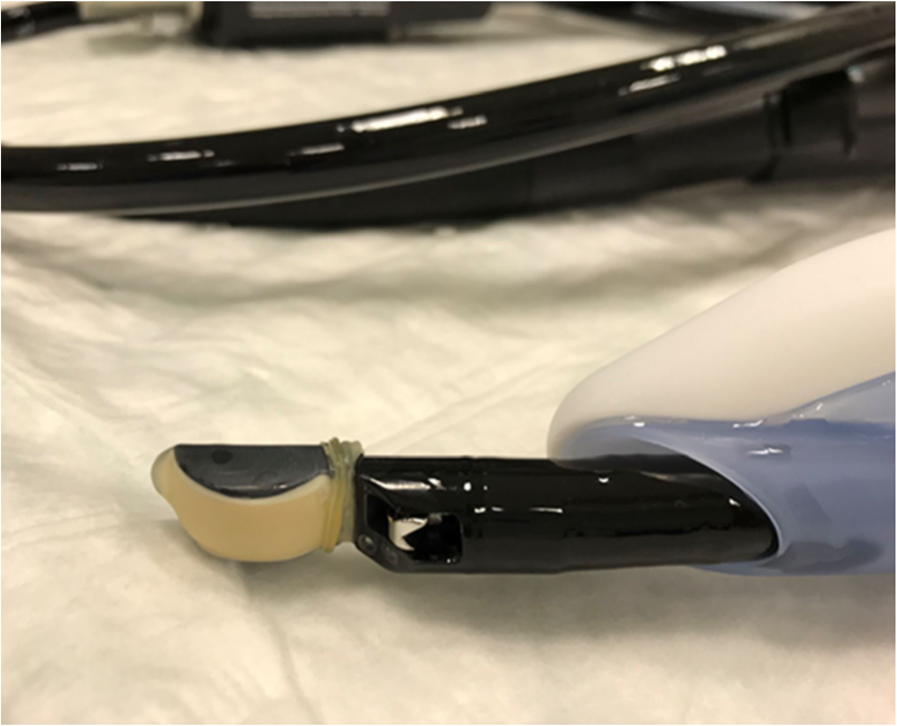
Endoscopic ultrasound probe passing through the LMA Gastro™


Procedures performed were endoscopic retrograde cholangiopancreatography (ERCP) (*n* = 7), peroral endoscopic myotomy (POEM) (n = 7) (Additional file 1: Figure S2), percutaneous endoscopic or fluoroscopic gastrostomy (PEG) (*n* = 5) (Fig. 2, Additional file 4), endoscopic submucosal dissection (ESD) and submucosal tunneling endoscopic resection (STER) (*n* = 6) or other advanced procedures (e.g. esophageal stent placement; Fig. 3; Additional file 2; endoscopic ultrasound probe, Fig. 4) in patients with considerable comorbidity (*n* = 6). The feasibility of the LMA Gastro™ for endoscopic intervention was rated as satisfactory by the four endoscopists (Table 1).

**Additional file 4.** Percutaneous endoscopic gastrostomy through LMA Gastro™. Access to the videos also via the following link: https://drive.google.com/drive/folders/1mVOhwgJGT5y5l5WE-cTyQpitMEc7VS88?usp=sharing.


**Fig. 5:**
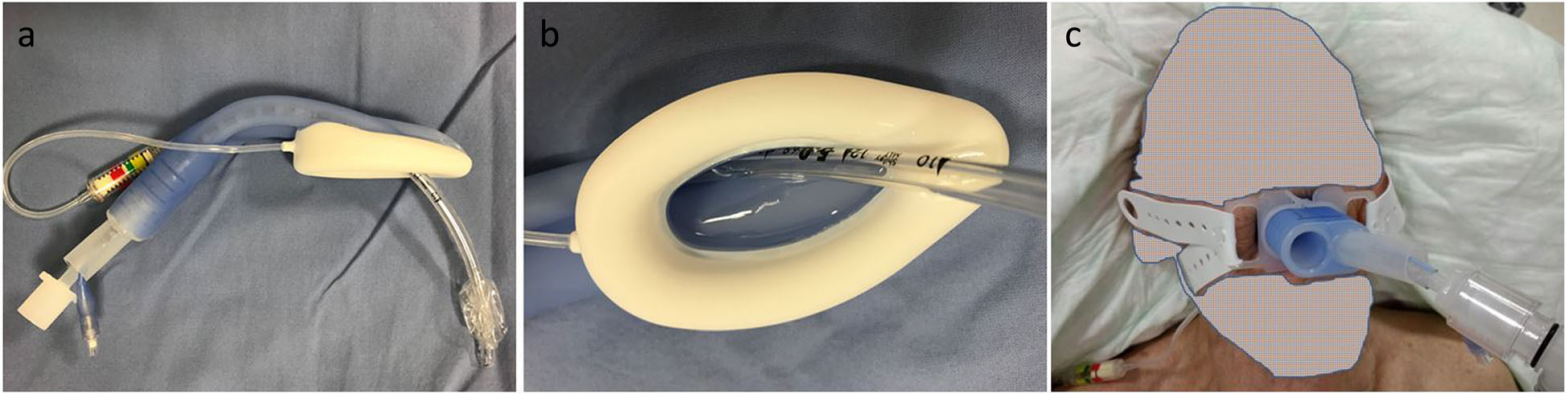
**a** Microlaryngoscopy tube (5.0 mm I.D., 6.9 mm O.D) passing through the airway lumen of the LMA Gastro™. **b** proximal end of the LMA Gastro™ with ETT in place. Please notice the large gastric channel narrowing the airway lumen. **c** LMA Gastro™ with a microlaryngoscopy tube in tracheal position

Placement of the LMA Gastro™ was reported as easy. All LMA Gastro™ were positioned at the first insertion attempt. In four patients, ventilation with LMA Gastro™ was not possible and tracheal intubation was performed instead. In one of these patients tracheal intubation was performed through the LMA Gastro™ using a 5.0 mm I.D. (6.9 mm O.D) microlaryngoscopy tube (Fig. 5 a-c; Additional file 3). The tube was mounted on a fiberscope (Ambu® aScope™ 4 Broncho Slim 3.8). In two other patients, one additional attempt at LMA Gastro™ placement was necessary.

Positive pressure ventilation was performed without any difficulties. There were no detectable differences regarding peak airway pressures or tidal volumes after insertion of the flexible endoscope. No signs of hypoxia, regurgitation or aspiration were noted. Even complex interventions with considerable insufflation of CO_2_ did not result in clinically significant hypercarbia.

One patient developed subcutaneous emphysema of the neck and left chestwall after endoscopic resection of an esophageal leiomyoma. This condition resolved spontaneously without any intervention.

There was not a single dislocation of the LMA Gastro™ due to endoscope movement through the designated channel.

### Post interventional course

There were no clinically significant adverse events within the cohort. No patient had to be admitted to the intensive care unit unexpectedly. All but one patient were discharged from PACU to a regular ward. We did not observe any pharyngeal bleeding. One patient developed a minor uvular hematoma, possibly due to positioning, and had difficulties swallowing for two days following the intervention.

## Discussion

In this retrospective analysis, we demonstrated the feasibility of the LMA Gastro™ airway as a valuable tool for advanced gastrointestinal endoscopic interventions under general anesthesia in high-risk patients.

The design of the LMA Gastro™ with its wide endoscope channel, an integrated bite block and an adjustable holder facilitates easy passage of a well lubricated endoscope (Fig. 2). The use of SAD usually reduces anesthetic dose requirements and decreases recovery time compared to tracheal intubation in nonendoscopic procedures. Furthermore, relaxation is not required. Compared to patients with orotracheal intubation, endoscopists estimated conditions with regard to access to the gastrointestinal tract as equal or better. The LMA Gastro™ aids the insertion of the gastroduodenoscope by guiding it into the upper esophagus. This may be the reason for the high degree of satisfaction we found in our setting.

We performed about 87% of all anesthetics for endoscopic intervention with the LMA Gastro™ successfully. Secondary conversion to an oral tracheal tube was required for four patients only, three of them with a history of major oral cancer and radiation therapy. Due to our experience, we would not recommend the use of LMA Gastro™ in this patient population because of the difficulties in positioning, risk of bleeding and an insufficient seal of the LMA with the periglottic structures.

The cuff of the LMA Gastro™ is more voluminous compared to first generation SAD [17]. Nevertheless, anesthesia providers reported insertion efforts similar to other second generation SADs. The pre-curved design and the bendability improved handling and enabled providers to insert the device in the supine and left lateral position without any difficulties. Providers should be cautious during insertion, as the distal opening of the endoscope channel may entrap soft palate structures causing mucosal lacerations.

The increasing popularity of deep sedation in patients undergoing upper gastrointestinal procedures will account for a rising number of anesthesiologist guided deep sedation. Patients who have previously showed poor tolerance to endoscopy under conscious sedation and who subsequently need to undergo prolonged and repeated procedures may be of particular benefit from the use of the LMA Gastro™. Intolerance of conscious sedation is a significant factor in failure of endoscopic procedures [18].

In advanced procedures like peroral esophageal myotomy (POEM) or endoscopic submucosal dissection (ESD), minimal patient movement is preferred to limit adverse events like perforation or bleeding.

ERCP is most often performed in spontaneously breathing patients under deep sedation without airway protection in the prone position, thereby reducing the risk of aspiration. However, patients with ASA physical status III and above, patients with severe cardio-pulmonary comorbidity, morbid obesity, obstructive sleep apnoea or reflux represent a group with an increased risk of cardio-respiratory adverse events [19, 20] and therefore tracheal intubation during general anesthesia is a common technique for this population [21].

Although studies of airway management using first generation “classic” LMA during ERCP in adults [22] and during esophagogastroduodenoscopy in paediatric patients [23] have been performed, this technique is not commonly used. This accounts to an even lesser extent for long interventions in high-risk patients. The LMA Gastro™ may offer a suitable alternative for airway control during ERCP.

An additional indication to use the LMA Gastro™ may be transesophageal echocardiography (TEE) under deep sedation. We used TEE in a patient with a left ventricular assist device immediately before ERCP.

On principle, the LMA Gastro™ was not designed to facilitate tracheal intubation. The narrow internal diameter (ID) of the airway lumen prevents a tracheal tube from passing through. If tracheal intubation through a SAD is planned, we therefore recommend to either exchange the LMA Gastro™ for another SAD that will allow direct passage of an adult-sized ETT or using a microlaryngoscopy tube, a pediatric-sized standard ETT (5.0 mm ID) with an adult length, mounted over a bronchoscope and inserted through the LMA Gastro™ into the trachea.

We are fully aware, that some of our patients met the criteria for tracheal intubation. Recommendations for patients with achalasia undergoing POEM typically include rapid sequence induction followed by tracheal intubation [24–26]. In our approach, patients with no residual solid food during prior diagnostic esophagoscopy, after induction of anesthesia without face-mask ventilation and insertion of the LMA Gastro™, the endoscopist immediately advanced the gastroscope through the gastric channel into the esophagus and stomach and evacuated any residual esophageal content.

This study has several limitations. First, the retrospective design at a single university medical center limits our conclusions, so they may not be generalizable. Second, the data relied on provider documentation to identify the use and success of the airway device. Due to the retrospective nature of this study, the reasoning behind a provider’s decision to perform tracheal intubation rather than use an SAD could not be assessed. Third, this is an observational study, not including a control group.

Our analysis should be considered as a pilot study for future randomized clinical trials with multicenter and prospective design to clarify the role of SAD devices during general anesthesia for endoscopic procedures. Especially comparisons with alternative techniques using other airway devices and monitored anesthesia care without airway protection are needed to evaluate the risks and benefits of different techniques.

In conclusion, we demonstrated the feasibility of the LMA Gastro™ during general anesthesia for endoscopic procedures in high-risk patients.

## Supplementary information

**Additional file 1: Figure S1**. CONSORT diagram of patient recruitment. **Figure S2**. Gastroduodenoscope with attachment cap for peroral endoscopic myotomy (POEM), passing through the gastric channel of a LMA Gastro™.

**Additional file 2.** Esophageal stent through LMA Gastro™. Access to the videos also via the following link: https://drive.google.com/drive/folders/1mVOhwgJGT5y5l5WE-cTyQpitMEc7VS88?usp=sharing.

**Additional file 3.** Microlaryngoscopy tracheal tube through LMA Gastro™. Access to the videos also via the following link: https://drive.google.com/drive/folders/1mVOhwgJGT5y5l5WE-cTyQpitMEc7VS88?usp=sharing.

## Data Availability

All data generated or analyzed during this study are included in this published article and its supplementary information files. Raw data are available upon reasonable request from the corresponding author.

## Abbreviations

ASA: American society of anesthesiologists
BMI: Body mass index
CO_2_: Carbon dioxide
ECG: Electrocardiogram
ERCP: Endoscopic retrograde cholangiopancreatography
ESD: Endoscopic submucosal dissection
ETT: Tracheal tube
F_I_O_2_: Fraction of inspired oxygen
ID: Internal diameter
LMA: Laryngeal mask airway
PEG: Percutaneous endoscopic or fluoroscopic gastrostomy
POEM: Peroral endoscopic myotomy
SAD: Supraglottic airway device
STER: Submucosal tunneling endoscopic resection
STROBE: Strengthening the Reporting of Observational Studies in Epidemiology
TEE: Transesophageal echocardiography

## Footnotes

1 LMA is a registered trademark of The Laryngeal Mask Company Ltd., an affiliate of Teleflex Incorporated
