# Supplementary material for: LMA Gastro™ airway is feasible during upper gastrointestinal interventional endoscopic procedures in high risk patients: a single-center observational study
Source: BMC Anesthesiol. 2020 Feb 8;20:40. doi: 10.1186/s12871-020-0938-9 (PMC7007643; doi:10.1186/s12871-020-0938-9)
Supplement: Supplementary file 2 — Additional file 1: Figure S1. CONSORT diagram of patient recruitment. Figure S2. Gastroduodenoscope with attachment cap for peroral endoscopic myotomy (POEM), passing through the gastric channel of a LMA Gastro™. [file 12871_2020_938_MOESM1_ESM.docx]

**Supplement**


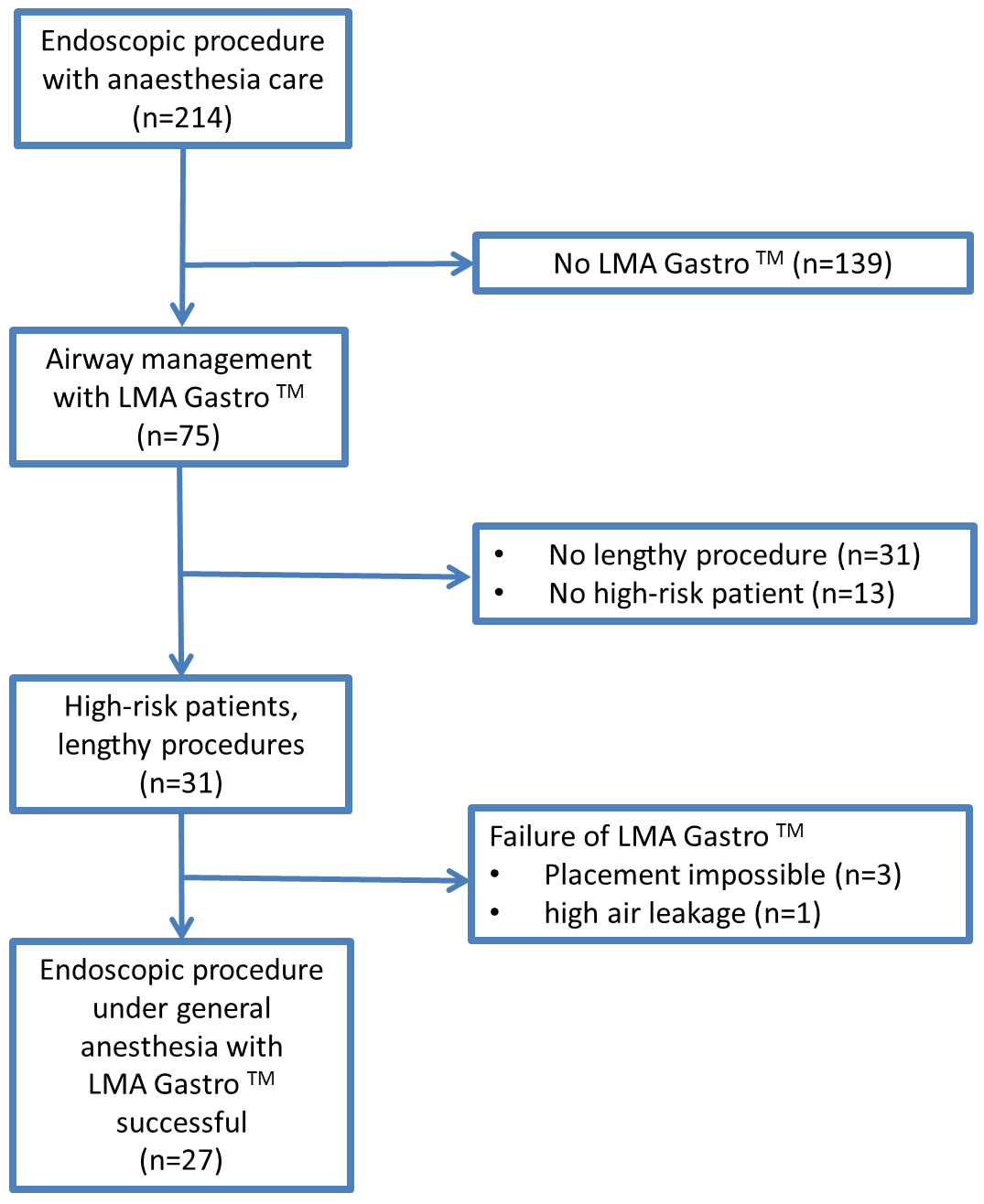


**Fig S1:** CONSORT flow diagram


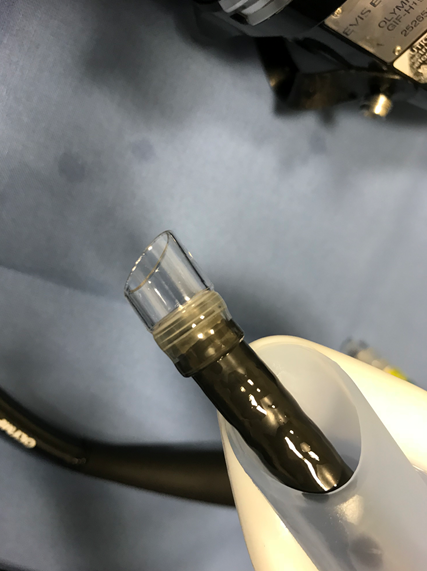


**Fig S2:** Gastroduodenoscope with attachment cap for peroral
endoscopic myotomy (POEM), passing through the gastric channel
 of a LMA Gastro^TM^
